# Copy number alterations analysis of primary tumor tissue and circulating tumor cells from patients with early-stage triple negative breast cancer

**DOI:** 10.1038/s41598-022-05502-6

**Published:** 2022-01-27

**Authors:** Marco Silvestri, Matteo Dugo, Marta Vismara, Loris De Cecco, Davide Lanzoni, Andrea Vingiani, Secondo Folli, Maria Carmen De Santis, Filippo de Braud, Giancarlo Pruneri, Serena Di Cosimo, Vera Cappelletti

**Affiliations:** 1 Department of Applied Research and Technological Development, Fondazione IRCCS Istituto Nazionale dei Tumori, Via Giovanni Antonio Amadeo 42, 20133 Milan, Italy; 2 Department Pathology and Laboratory Medicine, Fondazione IRCCS Istituto Nazionale dei Tumori, Via Giacomo Venezian 1, 20133 Milan, Italy; 3 Breast Cancer Unit, Fondazione IRCCS Istituto Nazionale dei Tumori, Via Giacomo Venezian 1, 20133 Milan, Italy; 4 Department of Radiotherapy, Fondazione IRCCS Istituto Nazionale dei Tumori, Via Giacomo Venezian 1, 20133 Milan, Italy; 5 Department of Medical Oncology, Fondazione IRCCS Istituto Nazionale dei Tumori, Via Giacomo Venezian 1, 20133 Milan, Italy

**Keywords:** Breast cancer, Cancer genomics, Computational biology and bioinformatics, Biomarkers

## Abstract

Triple negative breast cancer (TNBC) is characterized by clinical aggressiveness, lack of recognized target therapy, and a dismal patient prognosis. Several studies addressed genomic changes occurring during neoadjuvant chemotherapy (NAC) focusing on somatic variants, but without including copy number alterations (CNAs). We analyzed CNA profiles of 31 TNBC primary tumor samples before and after NAC and of 35 single circulating tumor cells (CTCs) collected prior, during and after treatment by using next-generation sequencing targeted profile and low-pass whole genome sequencing, respectively. In pre-treatment tissue samples, the most common gains occurred on chromosomes 1, 2 and 8, and *SOX11* and *MYC* resulted the most altered genes. Notably, amplification of *MSH2 (*4/4 versus 0/12, *p* < 0.01) and *PRDM1* and deletion of *PAX3* (4/4 versus 1/12, *p* < 0.01) significantly characterized primary tumors of patients with pathological complete response. All patients with paired pre- and post-NAC samples reported a change in post-treatment CNAs compared to baseline, despite they showed at least one common alteration. CNAs detected after treatment involved genes within druggable pathways such as EGFR, cell cycle process and Ras signaling. In two patients, CTCs shared more alterations with residual rather than primary tumor involving genes such as *MYC, BCL6, SOX2, FGFR4*. The phylogenetic analysis of CTCs within a single patient revealed NAC impact on tumor evolution, suggesting a selection of driver events under treatment pressure. In conclusion, our data showed how chemoresistance might arise early from treatment-induced selection of clones already present in the primary tumor, and that the characterization of CNAs on single CTCs informs on cancer evolution and potential druggable targets.

## Introduction

Triple negative breast cancer (TNBC) is an operational term, hopefully soon to be abandoned, which defines tumors on the basis of what they lack (*i.e.*, hormone receptors and HER2), rather than on their own characteristics^1^. TNBC has been increasingly recognized as a heterogeneous disease that exhibits substantial differences in terms of genomic and transcriptomic profiles^2,3^. The extreme heterogeneity of TNBC has led to difficulties in finding suitable molecular targets and has been reflected in the limited benefit from targeted therapies observed in clinical trials for unselected TNBC patients^4^. Therefore, most TNBC patients are still treated with chemotherapy, despite the fact that it is effective in a proportion of cases and only slightly improves the outcome^5^.

Although several studies focused on somatic variants of chemoresistant TNBC^6^, DNA copy number alterations (CNAs) remain still unexplored. Moreover, most genomic studies failed to match different tissue samples from the same case, hindering studies addressing CNA changes during treatment. Recently, bioinformatic tools have been released allowing to reliably infer CNAs from next generation sequencing (NGS) data obtained by targeted sequencing of routinely collected tumor specimens^7^. Thanks to such improvement, herein we report a homogenous consecutive series of early-stage TNBC patients undergoing neoadjuvant chemotherapy (NAC) and followed up after surgery with curative intent until progression. In addition to CNAs of primary tumor before and after anthracycline and taxane-based NAC, we also performed low-pass whole genome sequencing (lpWGS) of circulating tumor cells (CTCs) collected prior, during, and after treatment.

## Materials and methods

### Targeted next generation sequencing profile of tissue samples

Formalin-fixed paraffin-embedded (FFPE) macro-dissected specimens obtained during routine diagnostic biopsy and/or resection of primary tumor were used. Tumor cell percentage was evaluated by two pathologists (GP and AV) using hematoxylin/eosin-stained slides from adjacent sections. For all patients excluding those attaining a pathological complete response (pCR), *i.e.*, absence of invasive cancer in breast and node surgical specimens, both pre- and post-NAC tumor samples were sequenced and matched germline DNA was obtained from negative lymph nodes or breast normal tissue. DNA was extracted using GeneRead DNA FFPE kit (Quiagen, Hilden, Germany) and analyzed using the IonAmpliseq™ Comprehensive Cancer Panel (CCP) (ThermoFisher, Waltham, MA, USA), which cover all exons of 409 cancer-related genes. Raw sequencing data were mapped to the human reference genome (hg19) using the TMAP algorithm implemented in the Torrent Suite software version 4.4.3 with default parameters. Aligned bam files for matched tumor and normal samples were analyzed for copy number alteration (CNAs) calling using ad-hoc workflow implemented on Ion Reporter version 5.10.

### CTC collection, processing and molecular characterization

Blood samples (10 mL) collected in K_2_EDTA tubes were subjected to CTC enrichment with Parsortix™ (Angle plc, Guildford, UK) from blood draw. Enriched cells were harvested according to manufacturer’s instructions and fixed for 20 min at room temperature (RT) with 2% paraformaldehyde. Fixed samples were stained immediately or within 24 h from enrichments with phycoerythrin (PE)-labeled antibodies against epithelial markers EpCAM (clone HEA-125, Miltenyi Biotec, Bergisch Gladbach, Germany, working dilution 1:11 for 10 min at 4 °C), cytokeratins (pan cytokeratin clone C11, Abcam, San Francisco, CA, USA, and pan cytokeratin clone AE1/AE3, NSJ Bioreagents, San Diego, CA, USA, working dilution 1:10 for 10 min at RT) and EGFR (clone 423,103, Santa Cruz Biotechnology, Dallas, TX, USA, working dilution 1:11 for 10 min at 4 °C), and with allophycocyanin (APC)-labeled antibodies recognizing leukocytes and monocytes: CD45 (clone 5B1, Miltenyi Biotec, working dilution 1:11 for 10 min at 4 °C), CD14 (clone M5E2, BD Biosciences Pharmigen, San Diego, CA, USA, working dilution 1:20 for 10 min at 4 °C), and CD16 (clone 3G8, BD Biosciences Pharmigen, San Diego, CA, USA, working dilution 1:20 for 10 min at 4 °C). Nuclei were stained with 1 g/mL Hoechst 33,342 (Sigma-Aldrich, Saint Louis, MI, USA) for 5 min at RT. Labeled cells were analyzed using the DEPArray™ (Menarini Silicon Biosystems, Bologna, Italy) within 2 days from staining to visualize and recover single cells manually selected based on fluorescence labeling and morphology.

Selected epithelial (PE^+ve^/APC^−ve^) or double-negative (PE^−ve^/APC^−ve^) single cells were recovered for downstream molecular analyses. Recovered single cells and pools of white blood cells (WBC) were subjected to whole genome amplification employing the Ampli1 WGA kit (Menarini Silicon Biosystems, Bologna, Italy). Amplified-DNA quality was checked with the Ampli1—QC kit (Menarini Silicon Biosystems, Bologna, Italy), and a low-pass whole genome sequencing (lpWGS) for detecting CNAs was performed using the Ampli1-Low Pass kit (Menarini Silicon Biosystems, Bologna, Italy) for barcoded libraries preparation, followed by sequencing with the Ion Torrent Ion S5-system (ThermoFisher, Waltham, MA, USA), using the Ion530 chip according to the manufacturer’s instructions.

WGS sequences, passing quality control check performed on Ion Torrent software, were aligned to the human reference genome (hg19) using tmap aligner tool on Torrent_Suite 5.10.0. CNAs were predicted by using QDNAseq 11.0 with the following settings: minMapq = 37, window = 500 kb. “Gain” and “loss” calls were filtered out by residual (> 4 standard deviation, SD) and black list regions reported in ENCODE database^8^. Segmented copy number data of each sample were extracted starting from log2Ratio value.

Considering the evaluation of CNA profile, chromosome 19 was not considered due to its biased deletion associated with the high CG base percentage^9^. Samples were classified as aberrant if presented one of the following features:

- At least 1 genomic region with amplification/deletion > 12.5 Mb
- Sum of amplification/deletion of different genomic regions > 37.5 Mb.

### Ethical approval

The study was conducted according to the guidelines of the Declaration of Helsinki, and approved by the Institutional Review Board and Ethics Committee of Fondazione IRCCS Istituto Nazionale dei Tumori di Milano on February 19, 2013.

### Informed consent statement

Informed consent was obtained from all subjects involved in the study.

## Results

### TNBC study cohort

A total of 19 patients scheduled to anthracycline/taxane-based NAC were included in this analysis. Relevant demographic, treatment, tumor characteristics and available tissue and CTCs genomic profile are summarized in Table S1.

### Copy number alterations in pre and post-NAC primary tumors

**Figure 1:**
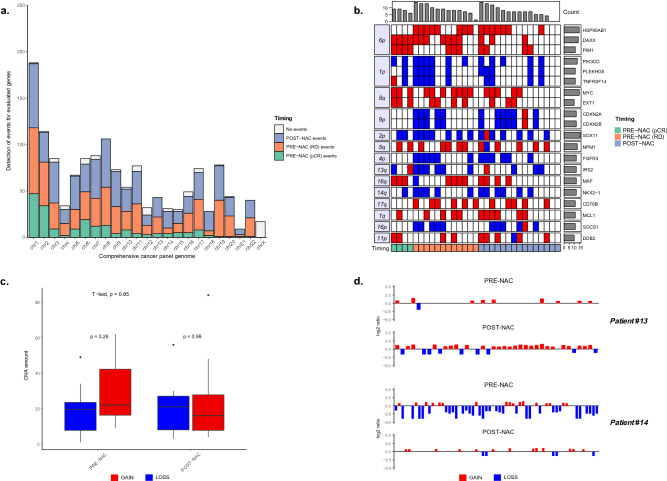
Copy number alteration (CNA) analysis of TNBC before and after neoadjuvant chemotherapy (NAC). (**a**) Barplot showing detection of CNA events obtained from all patients in the cohort. X-axis reports the genomic regions present in CCP panel divided by chromosomes while y-axis reports their absolute number of alterations by different sample type. “No events” refers to genomic regions with no alterations detected (**b**) Top 20 altered genes in the study cohort. The heatmap reports in the columns the pre- and post-NAC samples and in the rows the altered genes by chromosomal arms. Red and blue colors refer to amplification and deletion events respectively. (**c**) Box plot comparing pre- and post-NAC samples alterations (gain = red, loss = blue) in patients with matched tumors. (**d**) CNA plot for patients #13 and #14. Vertical lines represents the log2 ratio of gene copy number colored based on its status (amplification = red; deletions = blue).

Genomic alterations were evaluated in the initial diagnostic biopsies (n = 16) and surgical specimens of residual disease (n = 15); copy gains and losses across the entire genome were 744 and 630 in pre- and post-treatment samples, respectively (Table S2). The median number of CNAs per sample was 44 (range: 4–105), with amplifications (median: 23; range: 4–84) outnumbering deletions (median: 17; range: 0–56). The most common gains included chromosomes (chr) 1, 2 and 8, and *SOX11* and *MYC* were among the top-twenty altered genes (54% and 48% of cases, respectively), two findings consistent with previously reported literature data (Fig. 1a) ^10^.

When focusing on pre-treatment samples (mean cellularity = 70%), *HSP90AB1* (chr 6p), *CDKN2A/B* (chr 9p), *FGFR3* (chr 4p), *CD79B* (chr 7q), and *SOCS1* (chr 16p) were found unaltered in the four patients attaining a pathological complete response, possibly suggesting that these genes are important for maintaining a chemosensitive profile (Fig. 1b). Furthermore, gains of *MSH2* (chr 2p) and *PRDM1* (chr 6q) exclusively characterized the pCR patient group (4/4 versus 0/12, *p* < 0.01), and loss of *PAX3* (chr 2q) occurred in primary tumor samples of all patients with pCR and in a single case with residual disease at surgery (4/4 versus 1/12, *p* < 0.01) (Table S3). The network analysis of physical interactions between *MSH2, PRDM1 and PAX3* genes, *i.e.*, identified in responsive cases, reported a functional module of seven nodes including the mismatch repair, intrinsic apoptotic, Notch, Wnt, androgen receptor, HIF-1 signaling pathways, and platinum drug resistance (Figure S1). CNA events occurring in the paired pre- and post-treatment samples are summarized in Table 1.

**Table 1:** Summary of CNAs events in matched pre- and post-NAC samples.

Patient ID	Pre-NAC alterations (n)	Post-NAC alteration (n)	Shared alterations before and after treatment (%)
#1	53	61	42
#4	79	105	67
#5	26	7	7
#6	44	42	27
#10	36	15	6
#11	45	83	13
#12	63	35	31
#13	10	35	18
#14	51	18	14
#15	46	4	4
#16	66	52	14
#18	29	45	22

Overall, not a single case reported a post-treatment (mean cellularity = 73%) CNA profile identical to baseline; however, all cases had at least one common event in the paired analyses (Figure S2). The median number of CNAs following NAC was 45.5 in pre-treatment as compared to 38.5 of post-treatment samples (*p* = 0.85, Fig. 1c). More in detail, in 5 out of 12 (42%) patients, CNAs increased after NAC, with a median number of amplifications and deletions of 27 (range: 19–84) and 21 (range: 9–56), respectively (Table S4). Patient#13 who was chosen as a representative case for patients experiencing a post treatment increase in CNA, showed 35 CNAs in the residual tumor, 28 of which were newly acquired whereas 7 were shared with pre-treatment samples (Fig. 1d). In the remaining 7 women (58%), post-treatment CNAs dropped to a median number of 8 (range: 4–30) amplifications and 7 (range: 0–30) deletions. Patient#14 is a representative case of patients undergoing a decrease of genomic alterations after treatment, ending up with 18 events, eight of which already present in pre-treatment sample (Fig. 1d).

Since post-treatment samples are likely to be enriched in alterations associated with resistance that might be worth to target, we evaluated selected signaling pathways associated with actionable targets and found an enrichment for cell cycle process, EGFR and Ras signaling (Figure S3, Table S5).

### Copy number alterations in CTCs

In 10 patients (half of whom experiencing recurrent disease), we isolated 35 CTCs and matched leukocytes at different time points. The most frequent genomic alterations were on chr 9p, 10q, 10p and 22q (Figure S4, Table S6). From recurrent patient#13, three single CTCs collected at progression were analyzed and compared to primary and residual tumor tissues (Figure S5). CTC CNAs, including amplifications of *MYC, BCL6, DDR2, SOX2* and deletions of *CDKN2A/B*, were more likely to be shared with residual rather than primary tumor. Notably, one CTC showed its own distinct CNA profile, suggesting the emergence of a new or rather a previously-undetected clone at disease progression. Patient#6 had one CTC collected during follow up sharing to the same extend CNAs with both pre- and post-treatment samples including *LIFR*, *PIM1*, *RUNX1* amplifications (on chr 5, 6, and 21) and *FGFR4*, *FLT4* deletions (on chr5) (Table S7).

**Figure 2:**
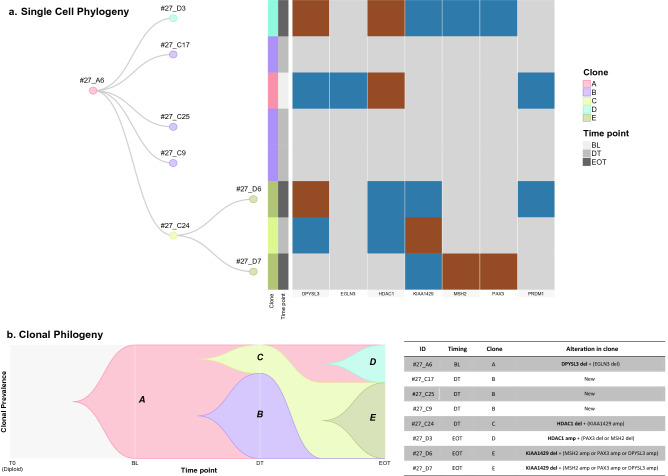
Phylogenetic analysis of patients#27. (**a**) Dendrogram and heatmap show single cell phylogeny obtained from TRONCO. Dendrogram (left) shows single CTCs colored by clones identified by TRONCO while heatmap (right) details the CNA alterations for each CTCs (rows) along the genes (columns) considered for the phylogenetic analysis. Brown and blue colors refer to amplification and deletion, respectively. (**b**) Representation of clonal phylogeny along time. the timescape plot (left) reports on Y-axis the prevalence of each clone while the table (right) describes clonal composition. The column “Alteration in clone” show CNAs defined as “priority” (bold) and “secondary” returned by TRONCO for each clone. The term “new” refers to set of clone with no alterations in genes considered for phylogenetic analysis.

Finally, for the index patient#27 we mapped the CTCs evolutionary relationship using the phylogenetic analysis performed by the TRONCO pipeline^11^. Patient#27 had 8 CTCs collected before (n = 1), during (n = 4) and after treatment (n = 3). Clone A (n = 1), was defined as the root of phylogenetic tree since it was detected at baseline (BL), clone B (n = 3) and C (n = 1) were identified during NAC treatment and were characterized by the absence of any alterations and deletion of *HDAC1* gene (priority), respectively. Beside to the loss of clone B, at the end of treatment two additional clones emerged: clone D (n = 1) and E (n = 2) characterized by the amplification of *HDAC1* (priority) and deletion of *KIAA1429* (priority) genes, respectively (Fig. 2). Therefore, treatment with NAC impacts on tumor trajectory, and the detection of CTCs with at least one CNA in common with their previous ancestors (clone D-A and E-C) and the loss of clone B at EOT, defined by no shared alterations with other clones, suggested a selection of tumor-driver events under treatment pressure.

To gain further insights into this hypothesis, we next examined differences in CNA events limiting to actionable genes retrieved from the OncoKB database (Table S8). Unique actionable alterations at the end of treatment included gains in the chromosomal regions harboring *MAP2K1*, *NTRK3*, *CDK12*, *ERBB2*, *PDGFR*, *RAF1*, and loss of regions containing *CDKN2A*, *JAK2*, *PTCH1*, *SMARCB1*. Overall, these results revealed that chemoresistant clones are enriched in cells with a distinct CNA profile, but also that there is frequent tiding of genetically different subclones. Ultimately, post-treatment CTCs show unique chromosomal alterations in actionable genes which might possibly inform on future treatment choices.

## Discussion

The clinical setting of NAC-treated patients represents the optimal model to study dynamics of clonal selection because the observational time from the start of treatment is short, *i.e.,*18–24 weeks), and it is thus unlikely that the clones emerging in the residual disease were not already present in the primary tumor.

In this hypothesis generating study, we showed that CNA profiles in the residual tumor only partially overlapped with the biopsy collected at baseline. We cannot exclude that such difference, although likely due to the effect of treatment, may at least in part be the consequence of tumor heterogeneity affecting the representativity of the biopsy with respect to the entire primary tumor. However, under selection pressure by the treatment, we hypothesize that specific clones, which were undetected in the initial bioptic sample, do instead emerge in the residual tumor possibly because they contain alterations enriched in treatment-resistant cells. Such findings lay the background for an early identification of resistant clones/genomic alterations associated with resistance with obvious implications for treatment planning. Thus, implementing longitudinal monitoring appears as a crucial step for improving NAC efficacy.

To get one step forward, and acknowledging both, the growing trend of last decades towards de-escalating surgical procedures not only on primary breast cancer but also on axillary nodes^10,12^, and the technical difficulties of sampling and processing different metastatic sites^13^, we hypothesized that liquid could replace tissue biopsies. Indeed, although rather preliminary, our data support the role of CTCs as a tissue-surrogate. Notwithstanding the still-to overcome technical difficulties in isolation and molecular characterization of single CTCs, we report proof-of-concept results showing that genomic alterations detected in single CTCs track the tumor evolution and reveal additional altered genomic regions beyond those already present in the residual tumor and which harbor genes recapitulating deregulated signaling pathways associated with resistance.

Although confirmatory studies on independent case series are needed, our results represent a novelty since so far only studies performed in the metastatic setting have explored CNA profiles in CTCs and tissue samples^12,14^, with only one study including a limited number of women (n = 11) with early-stage disease of different breast cancer subtypes^15^.

The reported findings only partially overlap with those we have previously reported on somatic variants and that were instead showing that besides cases with changed allele frequency (VAF) after NAC, there were a number of cases with stable mutant profile^6^. Thus, the different trend showed by CNAs and somatic variants suggested that these aberrations may provide complementary information on tumor biology.

We acknowledge two possible limitations in our study, one dealing with the reduced number of evaluable events which may be studied with targeted NGS. Nonetheless, the majority of chromosomal regions involved in TNBC were explored, as also indirectly supported by the good agreement with literature results^14^. The second limitation is represented by the low number of cases analyzed, and as such our results should be considered as hypothesis-generating rather than conclusive.

In summary, we show that tissue and CTC CNA analyses are informative on the treatment-induced clonal selection leading to chemo-resistance and represent an approach to be pursued in future studies to implement personalized medicine.

## Supplementary Information

Supplementary Information.

## Data Availability

Raw sequencing data are available from the corresponding author upon request.
