# Supplementary material for: Copy number alterations analysis of primary tumor tissue and circulating tumor cells from patients with early-stage triple negative breast cancer
Source: Sci Rep. 2022 Jan 27;12:1470. doi: 10.1038/s41598-022-05502-6 (PMC8795239; doi:10.1038/s41598-022-05502-6)
Supplement: Supplementary file 1 — Supplementary Information. [file 41598_2022_5502_MOESM1_ESM.zip › Supplementary_Materials&Methods_revised.docx]

**Supplementary materials and methods**

**Bioinformatic analysis**

CNAs data from each samples profiled with CCP were obtained using specific pipeline implemented on Ion Reporter software version 5.10. The CNAs status (amplification or deletion) obtained for each samples was called at single gene level. All the post-processing analyses were performed using R software (https://www.R-project.org).

For paired pre- and post-NAC analysis, clustering of genes according to binary CNAs status (amplification or deletion) was performed using unsupervised hierarchical clustering with Ward.D2 and Euclidean distance.

Pathway enrichment analysis on post-NAC samples was performed using the ClusterProfiler Bioconductor package considering 17 selected pathway from Gene Ontology (GO) biological process terms and KEGG pathways (Table S) [1]. Only enrichments with a pValue < 0.05 were considered.

The shared and private CNA events among tissue samples (pre- and post-NAC) and matched CTCs were derived from the intersection of whole genome data of CTCs (low-pass whole genome sequencing, lpWGS) with altered genes obtained from target gene sequencing of tissues. In particular, Genomic annotations associated with each genomic position in CTCs were retrieved using considering UCSC genome browser and Ensemble resources and hg19 as reference genome. Finally, CNAs obtained were defined as “shared” if characterized by the same type of alteration (amplification/deletion) in one of the following conditions:

- Both tissue samples of the same patients and all CTCs;
- Pre-NAC sample and at least one CTCs of the same patient;
- Post-NAC sample and at least one CTCs of the same patient.

The “private” label was assigned to CNA events never shared between CTCs and tissue samples of the same patients.

Fisher’s exact test were performed to retrieve CNAs distinguishing patients attaining pCR and cases with RD. For such a purpose, “amplifications vs non amplifications” and “deletions vs. non deletions” parameters were considered as input for the statistical test. Biogrid Human protein-protein interaction (PPI) network were considered to obtain connected functional module between genes retrived from the comparison between pCR and RD patients.

TraslationalOncology (TRONCO) pipeline with caprese algorithm, were used to perform phylogentic analysis on patient p27. In particular, the CNAs prioritization returned by TRONCO was used to identify and to map clonal relationship along time (Figure 1).

**Figure 1.** CNAs prioritization and phylogenetic analysis returned by TRanslational ONCOlogy (TRONCO) algorithm in patient p27. The tree reports relationship between genes status (amplification=red, deletion=blue) and their role for the reconstruction of the clonal phylogeny. Priority CNAs are reported on the top while the derived secondary alterations are shown on the bottom.

**List of R packages used:**

- Complexheatmap
- Pheatmap
- ClusterProfiler
- Igraph
- rTRM
- CNTools
- AnnotationHub
- TRONCO
- Cellscape
- Timescape

**Reference**

1. Yu G, Wang LG, Han Y, He QY. clusterProfiler: an R package for comparing biological themes among gene clusters. OMICS. 2012;16(5):284-287. doi:10.1089/omi.2011.0118
