# Supplementary material for: Copy number alterations analysis of primary tumor tissue and circulating tumor cells from patients with early-stage triple negative breast cancer
Source: Sci Rep. 2022 Jan 27;12:1470. doi: 10.1038/s41598-022-05502-6 (PMC8795239; doi:10.1038/s41598-022-05502-6)
Supplement: Supplementary file 1 — Supplementary Information. [file 41598_2022_5502_MOESM1_ESM.zip › Supplementary_data_revised.docx]

**Supplementary data**

**
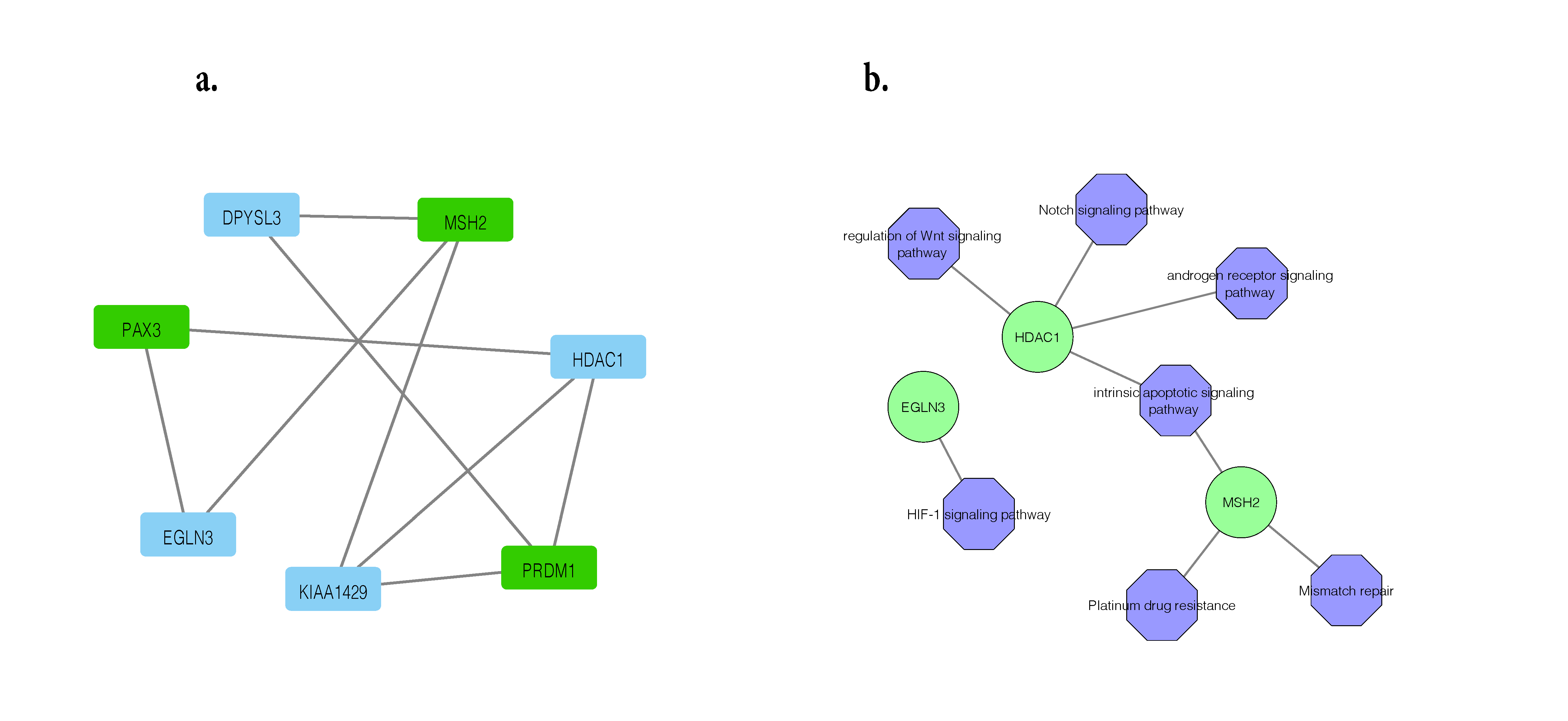
**

**Figure S1.**Functional interactions network analysis of genes differentially altered between patients attaining pathological complete response (pCR) and cases with residual disease (RD). (a) A network of 7 nodes were obtained starting from genes differentially altered between pCR and RD patients (highlighted in green) using Biogrid protein-protein interaction data. (b) Most representative targeted signaling pathways (represented in violet) were obtained from Gene Ontology (GO) and Kyoto Encyclopedia of Genes and Genomes (KEGG) databases.


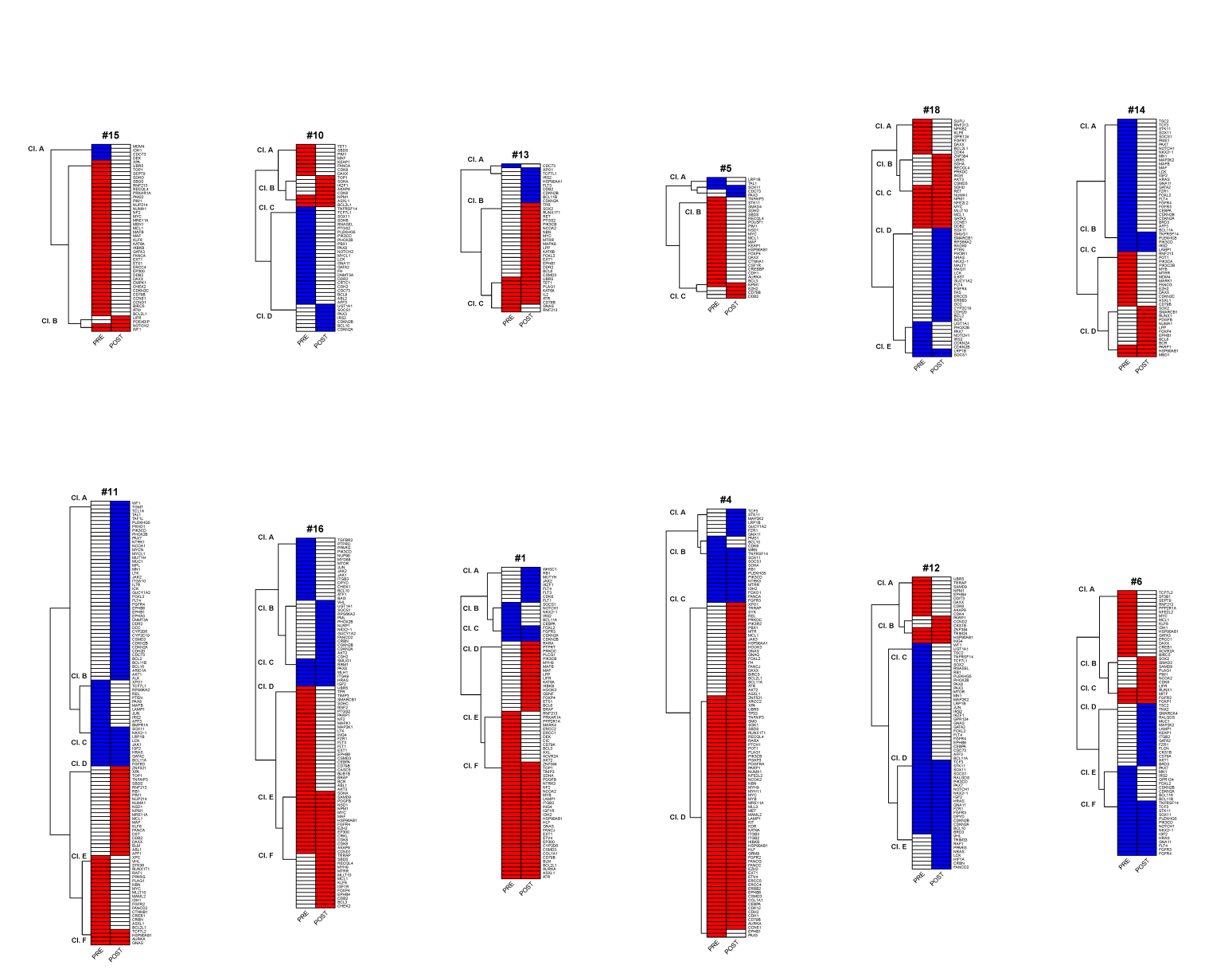


**Figure S2.** Assessment of CNAs distribution between matched pre- and post-NAC samples. Pre/post-NAC samples and genes were reported on the columns and on the rows, respectively. Alterations at pre- and post-NAC are grouped according to their status (amplification=red; deletions=blue) and the related cluster group ID were reported beside the dendrograms. Diploid genes in pre- and post-NAC samples for each patient were not reported.


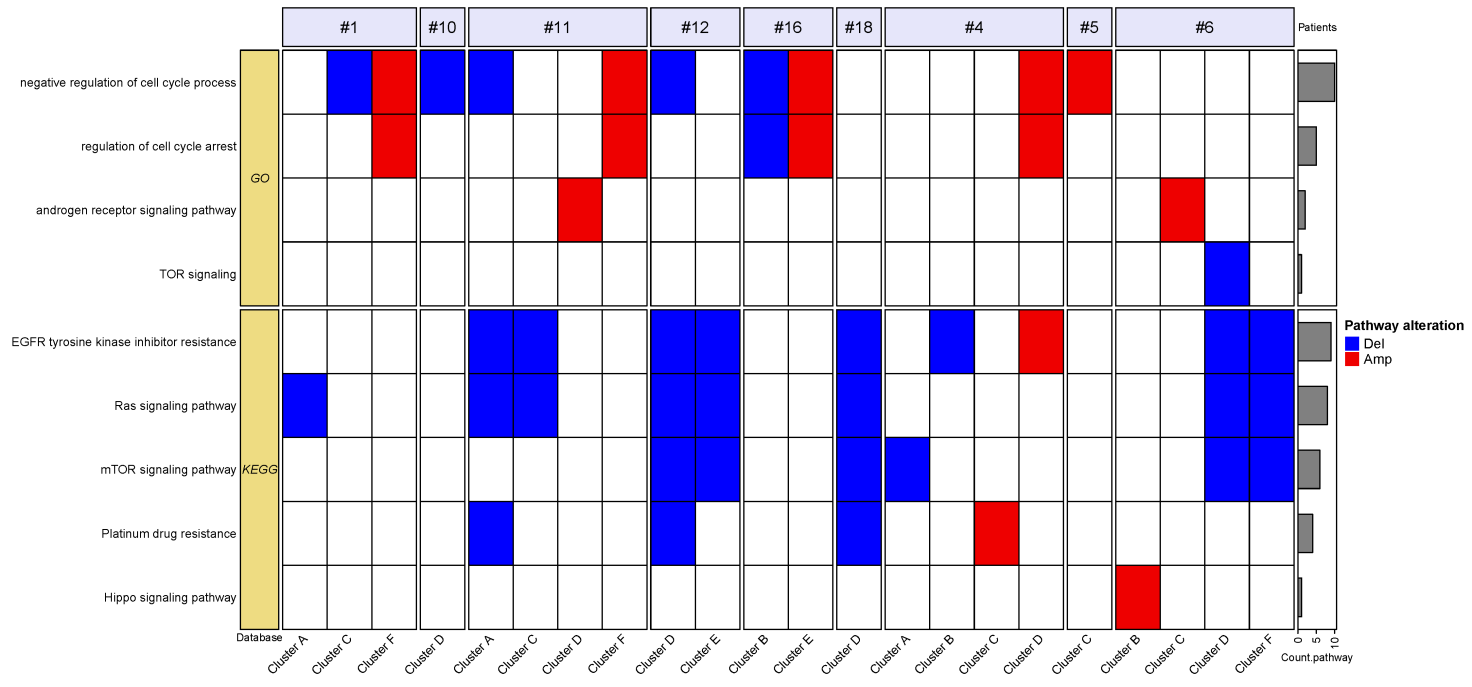


**Figure S3.** Pathway enrichment analysis of residual CNAs at post-NAC. The heatmap shows clusters of alterations at post-NAC for each patients on the columns and the altered canonical pathways (9/17) resulting from enrichment analysis on the rows. Red and blue colors refer to amplification and deletion, respectively.


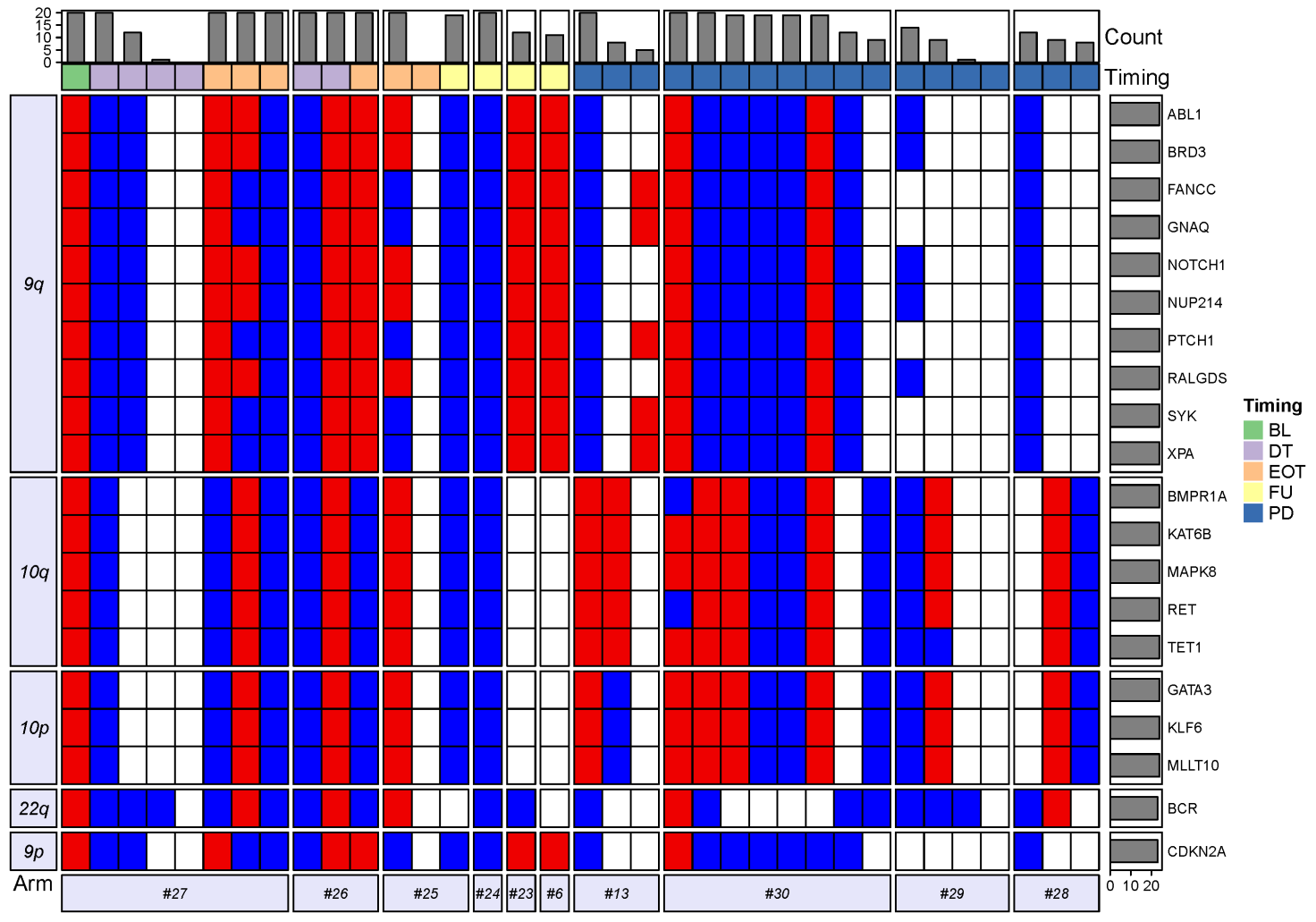


**Figure S4.** Top 20 altered genes in CTCs samples. The heatmap reports CTCs for each patient on the column and the altered genes divided by chromosomal arms on the rows. Red and blue colors refer to amplification and deletion events respectively. When possible, the CTCs of each patient were ordered based on the time of collection: baseline (BL), during treatment (DT), end of treatment (EOT), follow-up (FU) and progression (PD).






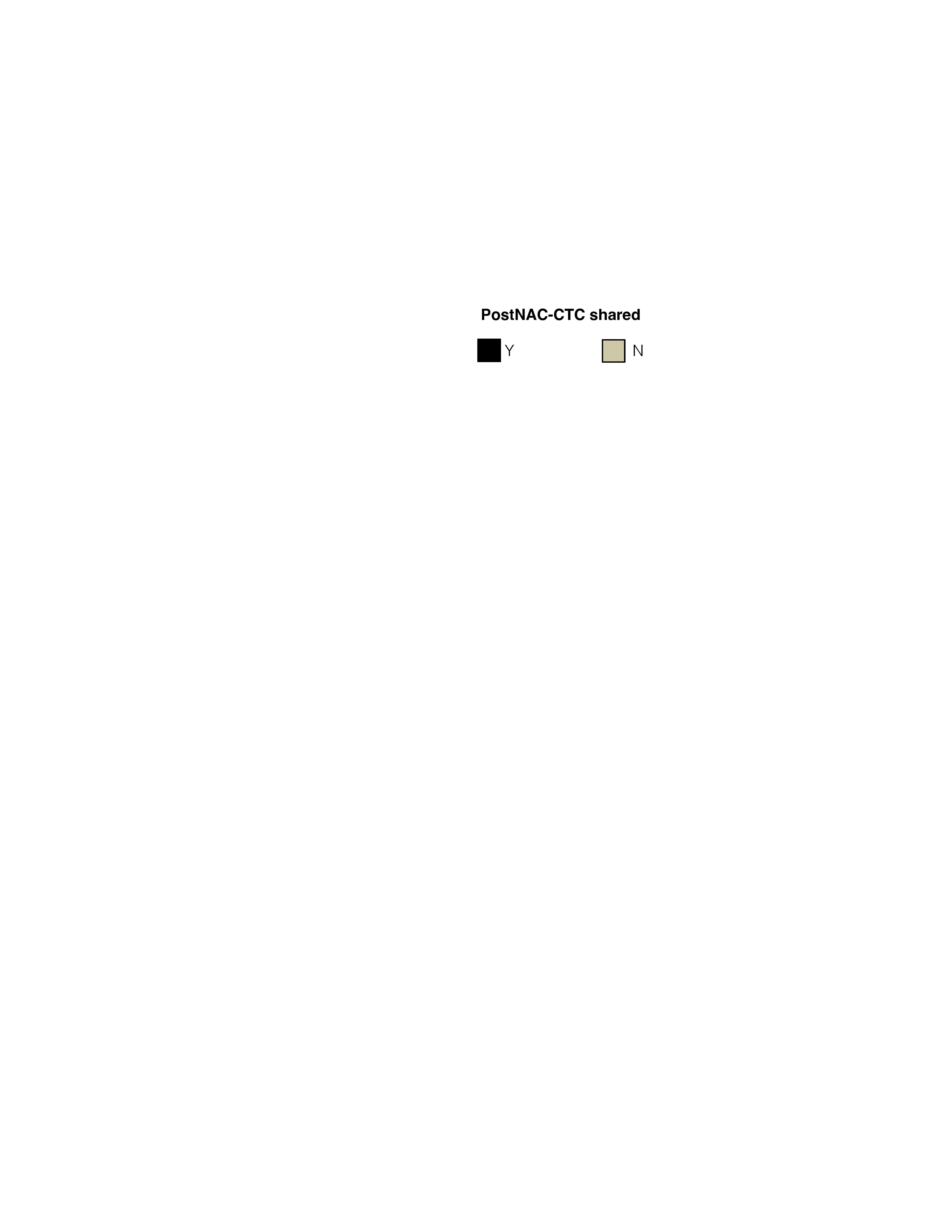


**Figure S5.** Comparison between tumor tissues samples and matched CTCs. The heatmaps show samples on the rows and genes derived by the intersection of tissue (CCP panel) and CTCs (lp-WGS) alterations on the columns. Red and blue colors refer to amplification and deletion, respectively. Color-bar on the top of the heatmaps reports shared (black) and non-shared (light-brown) CNAs events between post-NAC sample and at least 1 CTCs (see supplementary S6 for more details).
